# Continued need for non-human primate neuroscience research

**DOI:** 10.1016/j.cub.2018.09.029

**Published:** 2018-10-22

**Authors:** Anna S. Mitchell, Alexander Thiele, Christopher I. Petkov, Angela Roberts, Trevor W. Robbins, Wolfram Schultz, Roger Lemon

**Affiliations:** 1 University of Oxford, Department of Experimental Psychology, Oxford OX1 3SR, UK; 2 Institute of Neuroscience, Newcastle University Medical School, Newcastle upon Tyne NE2 4HH, UK; 3 Department of Psychology, University of Cambridge, Cambridge CB2 3EB, UK; 4 Department of Physiology, Development and Neuroscience, University of Cambridge, Cambridge CB2 3EB, UK; 5 Institute of Neurology, University College London, London WC1N 3BG, UK

## Abstract

Neuroscience research in non-human primates (NHPs) has delivered fundamental knowledge about human brain function as well as some valuable therapies that have improved the lives of human patients with a variety of brain disorders. Research using NHPs, although it is facing serious challenges, continues to complement studies in human volunteers and patients, and will continue to be needed as the burdens of mental health problems and neurodegenerative diseases increase. At the same time, research into the 3Rs is helping to ameliorate the harms experienced by NHPs in experimental procedures, allowing the effective combination of optimal welfare conditions for the NHPs and high quality research.

## Teaser

Mitchell *et al.* outline the benefits of non-human primate research for medicine and argue that the general public in the UK has been misled about the degree to which animals are harmed during research.

## Main Text

We write as a group of UK scientists committed to neuroscience research in NHPs at a critical point in the debate over their use in research. Our commitment stems from the notable advances that this research has already achieved, and because of our awareness that the monkey represents the best available model for our understanding of the human brain. Last year, the EU SCHEER report [1] highlighted key research areas where NHPs were still needed. These included testing of new drugs, infectious diseases (including Ebola, Zika and TB), and neuroscience. The need to alleviate the modern burdens of human neurodegenerative diseases and mental health disorders represents one of the most powerful arguments for sustaining primate research. This research now engages modern molecular, electrophysiological, behavioural and imaging technologies to identify the underlying mechanisms of disease, and some of it will lead to successful treatments.

We emphasise that NHP studies, using invasive methods, complement those in human volunteers and patients, using non-invasive methods such as brain imaging and computational modelling. For example, the Brain Prize in 2017 was awarded to three scientists in the UK who, respectively, discovered the neuronal mechanism of reward in monkeys, modelled this reward mechanism, and tested these models in human volunteers using fMRI [2]. Recent analysis shows that scientists who use NHPs in their research are also very active in using non-animal approaches to address current problems in basic and translational neuroscience [3]. NHP scientists also contribute to advances in welfare and the 3Rs [3].

Similarly, close interaction between human and monkey studies was also vital to the development of the parkinsonian model in the macaque which led to the discovery that deep brain stimulation of a tiny brain structure, the subthalamic nucleus, was an effective therapy for the motor disorders associated with Parkinson’s disease, research that was recognised by the Lasker Award in 2014 [4]. Over 250,000 Parkinson patients worldwide have since benefited from this treatment. Recent developments that have come directly from monkey research include the use of brain–machine interfaces for restoring movement to paralysed patients, new therapies for stroke patients with poor hand function, new strategies to harness brain plasticity to compensate for perceptual and cognitive impairment, and a better understanding of symptoms underlying psychiatric disorders as well as cognitive functioning in the healthy brain.

UK and EU animal research legislation requires a ‘harm–benefit’ analysis to determine whether the harms involved in the research are justified by advances in fundamental science or medical benefits that might result from the study. Therefore, in addition to assessing the benefits of NHP research, there has been an increased focus on reducing harms and understanding the overall level of severity experienced by research animals. This is of particular significance for NHPs in long-term neuroscience studies involving complex behavioural tasks.

Government regulators in the UK, and in some other EU countries, *prospectively* assess all such research as ‘severe’ because of the *potential* to cause severe harm. Critically, accumulating evidence shows that the lifetime experience of a great majority of the monkeys used in academic research in the UK is not severe, but is actually ‘moderate’. Starting in 2013, annual retrospective reviews of the *actual* severity experienced by each monkey in neuroscience research procedures has revealed that only 1% of the 366 monkeys used (New World marmosets and Old World macaques) actually experienced severe harms (as defined by the EU Directive 2013/63/), while most (66%) experienced moderate harms, or less (26% ‘mild’ and 7% ‘non-recovery’). These results are consistent with the broader set of United Kingdom Home Office statistics for basic research using NHPs over the same period (3% of severe procedures for the years 2013–2016). They also confirm the moderate level of harms that was first reported by the Home Office Pickard Report in 2013 [5]. This key distinction between the potential to cause severe harm and the actual level of severity is crucial but is often lost or misunderstood in discussions amongst the general public.

We are seriously concerned about calls to UK and other EU government regulators for a ban on all research involving NHPs, as well as in many other species, which has a prospective rating of severe. Such a ban would prevent disease-related medical and neuroscience research, damaging both fundamental and translational science. We have identified two serious consequences that such a ban would cause to NHP neuroscientific research; many more exist and will develop, impacting the wider scientific community, the welfare of non-human species, and human health.

*First*, it would ban most of the basic, fundamental neuroscience research, essential for understanding complex cognition and other processes that occur in the primate brain. We stress that the ban would result from the current situation in which most NHP neuroscience research is currently prospectively banded severe by government regulators, although as pointed out above, the great majority of primates used actually experience moderate harms or less.

*Second*, it would ban any present (or future) study in which a disease model is created in a monkey, indeed necessarily causing severe harms that reflect the impact of the brain disorder (e.g. Parkinson’s) in humans. But limited use of such models is still essential to help find effective treatments for a whole range of neurodegenerative and neuropsychiatric conditions that affect millions of humans.

We believe that in the coming period some increase in NHP studies in carefully targeted areas of neuroscience will still be needed. To ban useful primate disease models would be an extremely dangerous step to take, given the urgent need to find treatments for people suffering from these conditions.
